# Potential Genetic Markers Associated with Environmental Adaptability in Herbivorous Livestock

**DOI:** 10.3390/ani15050748

**Published:** 2025-03-05

**Authors:** Xiaotong Liu, Yongdong Peng, Xinhao Zhang, Wenting Chen, Yinghui Chen, Lin Wei, Qifei Zhu, Muhammad Zahoor Khan, Changfa Wang

**Affiliations:** Liaocheng Research Institute of Donkey High-Efficiency Breeding and Ecological Feeding, College of Agriculture and Biology, Liaocheng University, Liaocheng 252000, China

**Keywords:** herbivorous livestock, environmental adaptation, genome, candidate genes

## Abstract

Herbivorous livestock, such as cattle, sheep, goats, horses, and donkeys, play a crucial role in agricultural production and possess remarkable resilience to extreme environmental conditions, driven by complex genetic mechanisms. Recent advancements in high-throughput sequencing, genome assembly, and environmental data integration have enabled a deeper understanding of the genetic basis of their environmental adaptation. This review identifies key genes associated with high-altitude, heat, cold, and drought adaptation, providing insights into the molecular mechanisms underlying these traits. By elucidating these genetic adaptations, our study aims to support conservation efforts, inform selective breeding programs, and enhance agricultural productivity, ultimately contributing to sustainable livestock farming and economic benefits for farmers.

## Simple Summary

Environmental adaptation is a crucial factor for ensuring the health, survival, and productivity of herbivorous livestock. In recent years, researchers have identified several candidate genes that play a key role in climate adaptation, fundamental to the ability of herbivorous livestock to thrive under varying environmental conditions. This review aims to examine these genetic markers in detail, exploring their potential roles and impacts on various forms of environmental adaptation. The findings herein provide a scientific foundation for developing breeding strategies that can produce livestock breeds better equipped to face climate change.

## 1. Introduction

The climate and environment play a pivotal role in the long-term genetic evolution of species [1,2,3,4,5]. They not only act as core drivers of genetic variation but also have a profound influence on the formation and maintenance of biodiversity [6,7,8,9]. It has been well documented that environmental changes exert a wide range of effects on animal populations, which manifest not only in physical traits but also in the structure and function of tissues and organs, the regulation of physiological and biochemical processes, and gene expression [10,11,12]. Such adaptive responses enhance the ability of animal populations to thrive in changing environments, thereby reducing the risk of population extinction. Understanding how environmental factors mediate genetic variation and adaptation remains a central challenge in evolutionary genetics. Environmental changes, such as fluctuations in temperature, precipitation, and the availability of food and water, exert significant selection pressures on organisms [13,14]. These pressures drive genetic adaptations that enhance survival and reproductive success. Herbivorous livestock species, including cattle, sheep, horses, and donkeys, provide notable examples of such adaptations. Over extended evolutionary periods, these animals have developed remarkable resilience to fluctuations in environmental conditions, such as variations in oxygen levels, temperature, humidity, and water availability. Notably, these species are capable of thriving in extreme climatic conditions, including high altitudes, heat, cold, and drought. They have become essential providers of material resources for humans living in such challenging environments; for example, the goat, often referred to as the ’poor man’s cow’, is particularly known for its adaptability [15]. Investigating the genetic markers associated with these environmental adaptations is therefore critical for identifying the genes responsible for specific adaptive traits.

On the one hand, the growing emphasis on ecological and environmental conservation, combined with advancements in global climate monitoring technologies [16,17,18], has facilitated the collection and integration of extensive environmental data. These data, stored in detailed climate databases, include key climatic parameters such as temperature, humidity, water quality, and oxygen partial pressure [19,20]. These resources provide robust datasets for investigating the genetic basis of environmental adaptability in organisms [21]. On the other hand, the rapid development of next-generation sequencing technologies has enabled high-quality genome sequencing and assembly for a wide range of plant and animal species [22,23,24,25]. With the advantages of low cost, high throughput, and speed [26,27], next-generation sequencing (NGS) technologies have accelerated genomic research and laid the groundwork for identifying molecular genetic mechanisms underlying environmental adaptation in plants and animals [28]. As a result, the integration of genetic and environmental data has become a prominent area of research, attracting considerable attention from the scientific community worldwide.

By linking genetic data with environmental variables, researchers can more precisely identify genetic markers that are influenced by environmental factors. This is essential for understanding the molecular mechanisms of environmental adaptation [29,30]. Approaches commonly used for this purpose include whole-genome sequencing and the application of statistical methods to assess genetic differentiation (F_ST_), nucleotide polymorphisms (π), gene frequencies (Tajima’s D, ZHp, CLR, Hp), and measures of linkage disequilibrium (EHH, iHS, XP-EHH) [31,32,33]. These methods enable the identification of specific loci or genomic regions under environmental selection pressures.

The identification of genes associated with environmental adaptation is of paramount importance, particularly for the livestock industry. Enhancing the genetic resilience of livestock can have significant benefits, improving key economic traits such as production efficiency and reproductive success. Despite significant advancements in genomic technologies, there remains a critical gap in identifying the specific genetic mechanisms that enable local livestock breeds to adapt to extreme environmental conditions. Therefore, this review aims to provide an overview of recent advancements in the identification of genetic markers linked to environmental adaptations in cattle, sheep, goats, horses, and donkeys. By synthesizing current research, this article seeks to offer valuable insights and lay a strong foundation for future genomic studies in breeding, conservation, and molecular strategies aimed at improving animal welfare and ensuring sustainable livestock production.

## 2. Environment-Induced Adaptations in Herbivorous Livestock

Herbivorous livestock have developed a diverse array of environmentally induced adaptations, enabling them to survive and thrive in a variety of extreme habitats. These adaptations manifest across multiple levels, including morphological changes in appearance and growth patterns, structural modifications in tissues and organs, and shifts in blood physiological and biochemical parameters. Collectively, these adaptive traits underscore the complex interplay between genetic, physiological, and environmental factors, offering critical insights into the resilience and survival mechanisms of these animals. In this section, we will systematically explore these adaptations to uncover the underlying mechanisms that facilitate their environmental adaptability.

### 2.1. Appearance and Growth Characteristics

Livestock raised in high-altitude environments typically exhibit smaller body sizes, dense coats, and robust physiques, all of which contribute to enhanced disease resistance and stress tolerance. These traits are essential for survival in cold and oxygen-deprived environments at elevated altitudes, where they help mitigate environmental stressors such as low temperatures and reduced atmospheric oxygen [34,35,36,37]. In contrast, animals residing in hot environments generally possess lighter-colored and shorter coats, as well as smooth, thin skin. These features are advantageous for minimizing heat absorption and promoting efficient heat dissipation, thus enhancing their ability to survive extreme temperatures [38,39,40]. However, the challenging conditions in both cold, hypoxic high-altitude environments and hot, arid regions may lead to stunted growth, heat stress, reduced fertility, and increased susceptibility to infectious diseases. These factors can significantly influence the survival and reproductive success of these populations [41,42].

### 2.2. Morphological Adaptations of Tissues and Organs

Adaptations to high-altitude environments are notably reflected in the size and structure of vital organs, such as the heart and lungs, which are critical for survival in hypoxic conditions. In Tibetan sheep, for example, the lung-to-body weight ratio increases significantly as the altitude rises from 3500 m to 4500 m, while the heart-to-body weight ratio also exhibits a marked increase with higher elevations (2500, 3500, and 4500 m). Additionally, Tibetan sheep exhibit several key physiological adaptations, including increased pulmonary arterial volume, pulmonary arterial wall hypertrophy, enhanced elastic fiber content [43], and elevated alveolar density [44]. Similarly, the pulmonary architecture of yaks displays distinctive structural characteristics compared to lowland cattle, including an expanded alveolar surface area, attenuated alveolar septa and blood–gas barriers, and the presence of smooth muscle within microarteriolar walls (<50 μm in diameter), which collectively enhance gas exchange efficiency under hypoxic high-altitude conditions [45]. In extreme environments, adipose tissue accumulation serves critical functions in thermoregulation and energy reserve maintenance. A notable example is the fat tail of sheep, which enables these animals to endure periods of drought, food scarcity, and harsh climatic conditions [46]. Additionally, in arid regions, the increased dermal thickness of Kazakhstan’s white-headed cattle and the higher number of sebaceous glands in Kalmyk cattle enhance their resilience to extreme weather conditions [47].

### 2.3. Blood Physiological and Biochemical Indices

Blood physiological and biochemical parameters are crucial indicators of an animal’s adaptive responses to environmental stress. A study examining Baruwal sheep grazing at altitudes ranging from 2431 m to 3885 m revealed a significant increase in erythrocyte count, hemoglobin concentration, and erythrocyte pressure volume with increasing altitude [48]. Comparative studies on the hematological profiles of Tibet Alpine yaks (5100 m), Gannan yaks (3585 m), and Tianzhu white yaks (2960 m) demonstrated a significant elevation in red blood cell count (RBC), hematocrit (HCT), mean corpuscular volume (MCV), hemoglobin concentration (Hb), and mean corpuscular hemoglobin concentration (MCHC) in the Tibetan Alpine yaks, relative to the lower-altitude populations. These elevated blood indices reflect the adaptive response to hypoxic conditions, which is further supported by the increased activity of lactic acid (LA) and lactic dehydrogenase (LDH) [49].

In heat-tolerant livestock, such as N’Dama cattle (African trypanosome-tolerant) and White Fulani cattle (heat-tolerant), distinct differences in blood physiological indices have been observed. The White Fulani cattle exhibit significantly higher levels of albumin, hemoglobin, and mean hemoglobin content compared to the N’Dama cattle, which is believed to contribute to their superior heat tolerance [50]. Similarly, seasonal variations in blood indices have been reported in heat- and cold-tolerant sheep breeds, including Sirohi and Barbari (heat-tolerant) and Gaddi and Chegu (cold-tolerant). In general, hemoglobin (Hb), packed cell volume (PCV), total erythrocyte count (TEC), and total leukocyte count (TLC) were found to be higher in winter and lower in summer, with heat-tolerant breeds showing a less pronounced seasonal decline than cold-tolerant breeds. Furthermore, plasma enzyme levels (e.g., aspartate aminotransferase (AST) and alanine aminotransferase (ALT)), thyroid hormones (triiodothyronine (T3) and thyroxine (T4)), and blood glucose levels were significantly elevated in cold-tolerant breeds compared to their heat-tolerant counterparts [51]. Under drought conditions, dehydration leads to hemoconcentration and reduced blood volume in livestock, resulting in increased hemoglobin levels and erythrocyte pressure volume. Additionally, serum proteins, such as albumin, are elevated, although prolonged dehydration can cause a reduction in albumin levels and serum urea concentration [52].

## 3. Potential Genetic Markers Associated with Environmental Adaptation of Herbivorous Livestock

### 3.1. Genes Linked to Adaptations to High-Altitude Environments

High-altitude environments are characterized by hypoxic conditions, low temperatures, and increased ultraviolet radiation, all of which impose significant physiological challenges on the species inhabiting these regions [35]. The persistent selective pressures exerted by these harsh conditions have led to the evolution of specialized physiological traits in various species. These traits are often underpinned by specific genetic adaptations that enable organisms to survive and thrive in such extreme environments [53]. The adaptive mechanisms at high altitudes are primarily driven by selective genetic changes within the genome, which have been identified through studies of gene expression, polymorphisms, and molecular pathways associated with oxygen transport, thermoregulation, and cellular protection from oxidative stress.

A study examining the *EPAS1* gene, a key regulator of hypoxia responses, revealed evidence of positive selection in the genomes of several Tibetan domestic animals, including Tibetan goats, Tibetan horses, Tibetan sheep, Tibetan Mastiffs, Tibetan cattle, and Tibetan pigs. These findings provide valuable insights into the molecular mechanisms underlying adaptation to high-altitude conditions [11]. Ahmad et al. [54] further demonstrated that, in comparison to low-altitude cattle, several genes related to energy metabolism, oxygen transport, and body temperature regulation exhibited signs of positive selection in the yak genome. Notable examples include *CAMK2B*, which encodes a calcium signaling protein, and *GLUL*, a gene involved in nitrogen metabolism. These adaptations suggest that yaks are able to enhance nutrient absorption efficiency and adjust their energy metabolism to thrive under low-oxygen and high-altitude conditions [54]. Additionally, Lyu et al. [55] revealed a series of key genes through genome selection analyses that are closely associated with plateau adaptation, lipid metabolism, energy metabolism, immunity, and body size. These genes, introgressed into the genome of Tibetan cattle from yaks at varying altitudes, are involved in hypoxia response, cold adaptation, DNA damage repair, and ultraviolet radiation resistance, collectively enhancing the species’ response to high-altitude environments. Consistently, Terefe et al. [56] identified three new candidate genes (*CLCA2*, *SLC26A2*, and *CBFA2T3*) that are strongly selected in Ethiopian mountain cattle populations, along with the previously reported *ITPR2* gene. These genes are associated with the renin secretion KEGG pathway, ion channel activity, and response to hypoxia, which may play a crucial role in the adaptation of Ethiopian cattle to their high-altitude environment. The whole-genome analysis of Ladakhi cattle revealed a significant contribution from various genes to their adaptability in the high-altitude Himalayan environment. These genes primarily participate in processes related to hypoxia adaptation, energy metabolism, cold adaptation, and circadian rhythm. Notably, the interaction between genes associated with hypoxia signal transduction (*HIF-1*) and several enriched pathways (including PI3K, mTOR, NF-κB, ERK, and ER stress) plays a crucial role in constructing the complex adaptation mechanism of Ladakhi cattle under hypoxic stress conditions [57]. In addition, studies identified differentially expressed genes in the heart, lungs, and pulmonary vasculature of yaks and gayal, which contribute to high-altitude adaptation. Genes such as *UQCRC1*, *COX5A*, *CAPS*, *EDN3*, and *CHRM2* are involved in processes like oxidative phosphorylation, pulmonary vasoconstriction, and cardiac function, providing further evidence of genetic adaptations that optimize oxygen delivery and metabolic function at high altitudes. Notably, *EGLN1*, a key gene in the hypoxia-sensing pathway, was found to be downregulated across various tissues in both gayal and yak compared to lowland cattle, highlighting its role in facilitating adaptation to hypoxic environments [58]. Consequently, Wang et al. [59] reported that the expression of *ACSS2* in the liver of Tibetan cattle, residing at an altitude of 3600 m, was significantly higher than in Holstein cattle. This upregulation of *ACSS2* is thought to enhance metabolic efficiency via the HIF pathway, further contributing to the animal’s adaptation to hypoxic conditions. Similarly, Bao et al. [60] demonstrated that mitochondria is critical for adaptation to hypoxia. They identified several genes associated with hypoxic adaptation in the lungs, muscles, and spleens, including those encoding heat shock proteins (*HSPB7*, *HSPB2*, *HSPD1*, *HSPA1L*, and *HSP90AA1*) and genes involved in maintaining blood vessel density, such as *VEGFA*. In a separate study, Ge et al. [45,61] conducted two transcriptome studies on three types of yaks from varying altitudes (3400 m, 4200 m and 5000 m) and Zaosheng cattle at low altitudes. Their results suggest that the *UVSSA* gene (involved in DNA repair mechanisms) may play a critical role in the lung tissue of yaks, potentially facilitating their adaptation to the high levels of ultraviolet radiation at elevated altitudes. These findings offer valuable insights into the molecular mechanisms that underlie altitude-related environmental adaptation in yaks. Similarly, Guang-Xin et al. [62] identified seven candidate copy number variations (CNVs) from yak whole-genome sequencing data, which were associated with five key genes (*GRIK4*, *IFNLR1*, *GRHL3*, *LOC102275985*, and *LOC102275713*) and enriched in pathways related to hypoxic adaptation. Further genomic studies on yaks from both high-altitude and low-altitude populations revealed that four potential candidate genes, those being *RPS6KA6*, *ITPR1*, *GNAO1*, and *PDE4D*, are enriched in signaling pathways related to environmental information processing and adaptability [63]. These genes may play critical roles in helping yaks maintain homeostasis and optimize physiological functions in response to altitude-related stressors. Additionally, Qi et al. [64] observed that *EPAS1*, encoding HIF-2α, exhibited differential expression across various tissues in yaks from different altitudes. Specifically, *EPAS1* expression decreased in the heart but increased in the liver, muscle, spleen, and testes with altitude, suggesting its significant role in mediating adaptation to hypoxic environments. Whole-genome sequencing studies by Wang et al. [65] have revealed that CNVs in genes such as *DCC*, *GSTCD*, *MRPS28*, and *MOGAT2* contribute to the adaptation of yaks to high-altitude environments. In addition, previous studies documented matrix metalloproteinase-3 (*MMP3*) as a potential key target gene of hypoxia-inducible factor-1α [66]. Furthermore, Ding et al. [67] studied a polymorphism in the *MMP3* gene in high-altitude Pali yaks, which was present at a significantly higher frequency than in mid- and low-altitude populations. This mutation in *MMP3*, a target gene of HIF-1α, is believed to enhance the adaptability of yaks to the high-altitude environment by influencing extracellular matrix remodeling and vascular function.

Moreover, a comparison of Qaidam cashmere goats and Chengdu Brown goats using θπ and Fst values identified 1277 overlapping selection regions. Within these regions, genes associated with hypoxia and responses to elevated light intensity, such as *TH*, *ACER1*, *GNB1*, and *HIF1A*, were found to be enriched. This enrichment explains the adaptability of cashmere goats to the harsh conditions of the Tibetan Plateau [68]. In a related study, Jin et al. [69] used ClueGO functional analysis and Kobas analysis to establish a comprehensive high-altitude adaptation pathway network, identifying nine key genes—*LEPR*, *LDB1*, *EGFR* and *FGF2*—as critical for the high-altitude adaptation of Tibetan goats. Belay et al. [70] utilized the HP, Fst, and XP-EHH methods to identify a set of genes (*DDX28*, *RUNDC3B*, *PIK3CD*, *TIGAR*, *PTPMT1*, and *STXBP4*) associated with adaptation to hypoxic conditions in Ethiopian goats residing at high altitudes. Notably, *PTPMT1* has been identified as a key gene contributing to survival in these environments [71]. In a comparative study, Zhong et al. [72] compared low-altitude populations (Taihang goats at 600 m and Jining gray goats at 39–65 m) with high-altitude populations (Tibetan goats at 4000–4500 m). Using the Fst and XP-EHH indices, they identified the *ADIRF* gene as potentially beneficial for the adaptation of Tibetan goats to high altitudes. Moreover, Li et al. [73] discovered a 233 bp deletion in intron 7 of the *PAPSS2* gene. The frequency of this homozygous deletion was found to be 80.25% in Tibetan goats compared to just 28.75% in low-altitude goats. This suggests that *PAPSS2* may negatively affect the adaptation of Tibetan goats to the hypoxic environment of the Qinghai–Tibet Plateau. Interestingly, it was revealed that the unique allele of *PAPSS2* in Tibetan goats originated from a wild caprid species. Similarly, Sasazaki et al. [36] demonstrated that the frequencies of mutant alleles for two single-nucleotide polymorphisms (FGF5 c.-253G > A, EPAS1 Q579L) in Nepalese goats increased with altitude, a trend supported by the Pearson correlation coefficient. Additionally, studies have shown that the *FGF5* gene plays a critical role in hair growth [74], with long hair providing an advantage for survival in cold, high-altitude environments. The *EPAS1* gene, a transcription factor involved in various hypoxic adaptation mechanisms, also contributes to this adaptability. Therefore, polymorphisms in these two genes are crucial for understanding the genetic basis of high-altitude adaptation in Nepalese goats. Through the detection of genomic structural variations in Tibetan sheep and Hu sheep, Liang et al. [75] identified three specific structural variations (*EPAS1*, *PAPSS2*, and *PTPRD*) that are exclusive to Tibetan sheep. Haplotype construction revealed that these three structural variations are associated with altitude, demonstrating a significant correlation among them. Additionally, the study found that the expression of several genes closely related to cardiovascular function, including *BRINP3*, *CDH18*, *BDH1*, and *CYP2J*, was upregulated in response to hypoxic conditions. This finding further substantiates the notion that Tibetan sheep have undergone adaptive adjustments to thrive in high-altitude environments [75]. Meanwhile, Li et al. [76] analyzed transcriptome data from twelve organs (including the heart, liver, spleen, lung, kidney, and muscle) of both high-altitude Tibetan sheep and low-altitude Hu sheep. Their findings revealed that at least three genes—*HBBC*, *HBB*, and *EGLN1*—are differentially expressed in individual organs, thereby shedding further light on the genetic foundation of high-altitude adaptation in Tibetan sheep. Moreover, Zhao et al. [77] found that mutations in HIF-1α led to a series of changes in blood gas indicators in both Tibetan sheep and Gansu Alpine Merino sheep. Specifically, hematocrit (Hct), hemoglobin (Hb) concentration, the partial pressure of oxygen (PO_2_), and oxygen saturation (SaO_2_) were significantly lower than those in Hu Yang sheep. Furthermore, the half-saturated oxygen partial pressure (P50) in Tibetan sheep was significantly higher compared to Hu sheep, while the glucose (Glu) concentration was notably lower, enhancing the oxygen utilization capacity of Tibetan sheep.

Continuing in this line of research, Zhang et al. [78] utilized resequencing data from three sheep breeds to identify several genes—*HIF1A*, *ATR*, *SLC24A4*, *PPA2*, and *ROCK2*—associated with high-altitude adaptation in Tibetan sheep through selective sweep analysis. Notably, changes in mean corpuscular hemoglobin and elevated hemoglobin concentrations further suggest an adaptation to high-altitude environments. Additionally, resequencing data from 15 Tibetan sheep populations at varying altitudes, as reported by Liu et al. [79], revealed that high-altitude Tibetan sheep with deletion variants in the *HAG1* gene exhibited significantly higher body weight, body temperature, pulse interval, and levels of erythrocyte hemoglobin and globulin compared to low-elevation Qinghai Tibetan sheep lacking such variants. Furthermore, the expression of the *HAG1* gene was elevated in the liver and lung tissues of high-altitude Tibetan sheep, indicating that these deletion variants in the *HAG1* gene play a role in facilitating adaptation to high-altitude environments. Consistently, Wang et al. [80] identified a single-nucleotide polymorphism (SNP) downstream of the *CYP17* gene in Tibetan sheep. They found that the frequency of the dominant allele of this candidate gene increased with the altitude of the habitat across five sheep breeds. This finding supports the idea that mutations in the *CYP17* gene enhance the adaptive capacity of Tibetan sheep to hypoxic environments. Previous studies have also shown that the *CYP17* gene regulates hemoglobin levels and red blood cell mass and is associated with high-altitude polycythemia [81].

Recent studies have significantly advanced our understanding of genetic adaptation to high-altitude environments in various equid species. For instance, Han et al. [82] identified several genes associated with high-altitude adaptation in Ganzi horses, including *EPAS1*, *ABTB2*, *RHOQ*, and *TMEM247*, through comparisons with low-altitude horses. Notably, missense mutations in *EPAS1* (A > T) and *OR52A1J* (C > T) were found to be more prevalent in high-altitude horses, potentially contributing to the successful survival of Ganzi horses on the Tibetan Plateau. Additionally, Liu et al. [53] conducted the first comprehensive genome-wide analysis of 17 Chinese native horse breeds across varying altitude gradients. Their study revealed that *EPAS1* exhibited the strongest positive selection in Tibetan horses, with two missense variants within the PAS domains of the *EPAS1* gene significantly correlated with altitude. Tibetan horses residing at altitudes exceeding 4000 m showed the highest allele frequency for these variants (approximately 0.8), while the frequency in low-altitude horses (below 1000 m) was less than 0.05. These findings suggest that *EPAS1* is a critical gene for high-altitude adaptation. Furthermore, Liu et al. [83] investigated the survival strategies of Tibetan wild asses in the challenging environment of the Tibetan Plateau, focusing on their gut microbiota. Their results revealed that the intestinal microbiota of Tibetan wild asses undergoes differentiation and phenotypic alterations that enhance the host’s ability to thrive on sparse, low-quality forage. Macrogenomic analyses further identified the significant enrichment of plant biomass-degrading microbiota, alongside genes (*GH43*, *GH3*, *GH31*, *GH5*, and *GH10*) and enzymes (β-glucosidase, xylanase, β-xylosidase, etc.) involved in carbohydrate metabolism, thereby highlighting the adaptive role of the gut microbiome in supporting the Tibetan wild ass’s high-altitude survival. Additionally, Zeng et al. [84] reassembled the genome of an individual Kiang and analyzed the genomes of five Kiangs and 93 donkeys, including 24 Tibetan donkeys. Their investigation of high-altitude adaptation mechanisms in Kiangs and Tibetan donkeys revealed that the EPAS1 locus exhibits strong selective pressure in Kiangs, while the *EGLN1* gene plays a crucial role in Tibetan donkeys’ adaptation to low-oxygen environments. In a previous study, genome-wide selective signal analyses of Qinghai donkeys—inhabiting altitudes of 3000–5000 m—revealed very low ZHp values (<–7) and very high di values (top 1%). This analysis identified overlapping regions and two genes, *HBB* and *GLDC* [85,86], which have previously been associated with high-altitude adaptation, providing valuable molecular insights into the adaptation of Qinghai donkeys to low-oxygen conditions [87]. The potential genes associated with high-altitude adaptation in livestock are summarized in Table 1.

### 3.2. Genes Linked to Adaptations to Cold Environments in Herbivorous Livestock

The ability to withstand cold exposure is critical for the survival of herbivores in cold regions, where long, harsh winters impose significant challenges to metabolic processes, reproduction, and overall productivity. Understanding the molecular mechanisms underlying cold adaptation is essential for improving the resilience of livestock in cold climates, particularly in northern China and other regions with severe winter conditions. To gain a deeper understanding of the genetic basis of cold adaptation in livestock, various genetic approaches have been employed.

By constructing single-nucleotide polymorphism (SNP) and haplotype localization trees for the *ZC3H10* gene, and by combining FLK and hapFLK analysis methods, Wang et al. [88] found that all four cattle breeds inhabiting cold zones exhibited strong positive selection signals. This finding suggests that these cattle breeds may have developed adaptations to cold environments over a long evolutionary period. To further validate the function of the *ZC3H10* gene, we created a *ZC3H10* knockout fibroblast cell line. The experimental results indicated that the deletion of *ZC3H10* disrupted signaling pathways associated with thermogenesis and immune function, providing robust evidence for the significant role of *ZC3H10* in bovine adaptation to cold environments. A genome-wide selection analysis and an in-depth study of penetrance signaling in crossbred cattle have identified several key candidate genes closely associated with adaptation to environmental changes, the maintenance of metabolic homeostasis (including *TRPM8*, *NMUR1*, *OXR1*, *PRKAA2*, and *SMTNL2*), and immune responses (e.g., *PLCB4* and *SIN3A*). This research reveals the genetic mechanisms that enable crossbred cattle to adapt to cold climates [89]. While, Huang et al. [90] have newly identified a group of key candidate genes involved in heat production and energy metabolism, such as *UQCR11*, *DNAJC18*, *EGR1* and *STING1*, on chromosome 7 by differentiating genes within the selected regions of cattle herds in southern and northern China. These findings provide valuable information for an in-depth understanding of bovine adaptation to cold environments. Correspondingly, a genome-wide scan of northern and southern cattle populations was conducted to identify candidate genes involved in thermogenesis-related pathways [91]. Furthermore, they revealed through functional analyses that a variant in the *PRDM16* gene (c.2336 T > C, p.L779P) in northern cattle was involved in cold adaptation. This gene plays a crucial role in regulating the differentiation and function of brown adipocytes, influencing thermogenic capacity, and thus facilitating adaptation to cold environments [91]. Interestingly, a unique missense mutation was found in the highly conserved *NRAP* gene in the Yakutia breed [92]. This mutation was shared with 16 species of hibernating or cold-adapted mammals from at least six different phylogenetic orders. Furthermore, they suggested that various mammalian species may experience similar selective pressures in cold environments, leading to analogous mutations in the *NRAP* gene, which likely enhance adaptation to these conditions [92]. In addition, a selective scan and analysis of cold-adapted genes in Yanbian cattle was conducted by Shen et al. [93], who employed three methodologies to identify three candidate genes: *CORT*, associated with cold stress; *FGF5*, involved in hair follicle development and length; and *CD36*, which plays a crucial role in fat digestion and absorption. These findings provide new insights into the adaptations of Yanbian cattle [93]. Similarly, Ghoreishifar et al. [94] used de-correlated composite of multiple signal (DCMS) and adjusted *p*-values (FDR < 5%) from multiple testing to identify significant genomic regions. This analysis revealed genes such as *AQP3*, *AQP7*, and *HSPB8*, which are associated with cold adaptation in Swedish cattle. The Inner Mongolian Sanhe cattle demonstrate remarkable adaptability to extremely low temperatures, along with disease resistance and productivity. Hu et al. [95] observed significant elevations in the blood concentrations of adrenocorticotropic hormone (ACTH), triiodothyronine (T3), and thyroxine (T4) in Sanhe cattle exposed to −32 °C for 3 h [95]. Furthermore, the expression of the *HSP70* gene was significantly upregulated by *AQP3*, *AQP7*, and *HSPB8.* They found that a total of 20 SNPs were identified in the 5′-flanking regions of the *HSP70* gene in Sanhe cattle. Among these, SNP-42−, SNP-105+, SNP-181+, and SNP-205+ were significantly correlated with T3, while SNP-105+ was significantly correlated with T4. These findings suggest that the *HSP70* gene may play a key role in the cold acclimatization of cattle [95]. Consequently, Weldenegodguad et al. [96] identified a range of candidate selective sweep genes in cattle breeds exposed to cold environments. Notably, genes such as *DNAJC28*, *HSP90B1*, *AGTRAP*, *TAF7*, *TRIP13*, *NPPA*, and *NPPB* were identified in Eastern Finncattle, while *CD14*, *COBL*, *JMJD1C*, *KCNMA1*, *PLA2G4*, *SERPINF2*, *SRA1*, and *TAF7* were identified in Western Finncattle. Similarly, in Yakutian cattle, genes like *DNAJC9*, *SOCS3*, *TRPC7*, *SLC8A1*, *GLP1R*, *PKLR*, and *TCF7L2* were identified based on whole-genome sequencing data [96]. Similarly, a candidate region on Siberian cattle chromosome 15, identified through both a genome-wide association study (GWAS) and selective sweep scans, contains the *MSANTD4* and *GRIA4* genes, which are functional candidates for cold stress resistance due to their roles in the cold shock response and body thermoregulation, respectively [97].

The genetic mechanisms underlying thermoregulation and adaptation to cold environments have been extensively studied in sheep. In a related study, Ji et al. investigated the mechanisms of heat production in cold-exposed Altai lambs. Their findings revealed that the activation of the peroxisome proliferator-activated receptor (PPAR) signaling pathway, along with the calcium signaling pathway, in muscle tissues, contributed to enhanced thermogenesis [98]. In addition, this led to the upregulation of genes such as *APOC3*, *FABP4*, *LPL*, *PCK1*, *PLIN1*, *CABP1*, and CALN1. In contrast, in bearded lambs, the activation of cAMP signaling resulted in the downregulation of *ADCY10* and *ADORA2A*. These findings highlight the different thermogenic responses to cold exposure in muscle tissues of different breeds of lambs [98]. Furthermore, five genes in muscle tissue exhibited higher expression levels in Altai lambs compared to Hu lambs, indicating that muscle contraction in colder environments aids in the maintenance of body temperature (*BDNF*, *PVALB*, *TNNC1*, *MYL2*, and *ACTC1*). Accordingly, Jiao et al. [99] systematically compared the liver transcriptomes of Altai lambs, which are native to cold, arid climates, with those of Hu lambs, which originate from warmer, more humid regions. Their comprehensive analysis revealed that eight genes involved in muscle shivering thermogenesis, including *ACTA1*, *MYH1*, *MYH2*, *MYL1*, *MYL2*, *TNNC1*, and *TNNT3*, were significantly upregulated in the Altai lambs subjected to cold exposure. Additionally, genes linked to muscle non-shivering thermogenesis, such as *ATP2A1*, *SLN*, and *CKM*, exhibited enhanced expression, thereby contributing an overall increase in heat production and thermoregulatory efficiency in these lambs under cold stress conditions [99]. The subsequent transcriptomic analysis of the hypothalamus, caudal fat, and perirenal adipose tissues revealed that cAMP and calcium signaling pathways were significantly activated in response to cold exposure. This activation was associated with the differential expression of key thermogenesis-related genes, such as *UCP1*, *RYR1*, *ADIPOQ* and *LPL*, which were implicated in both species (Altay and Hu ewe lambs) studied [100]. In a related study, Saravanan et al. [101] identified an SNP (rs428553319) located in the 6.5–7.0 Mb region of chromosome 1 in Changthangi sheep, a breed known for its adaptation to cold environments. This SNP is under strong natural selection and affects several genes with potential roles in thermoregulation. Among these, *TRPM8*, a thermoreceptor involved in both cold and heat sensation, plays a crucial role in thermoregulation, pain perception, the inflammatory response, and vascular smooth muscle function [102]. Further studies have shown that during adaptation to the cold climate of Siberia, significant selective pressure was observed in a genomic region near the *ADAMTS5* gene across three sheep breeds: Kulundin, Altai Mountain, and Baikal [103]. Research has demonstrated that *ADAMTS5* knockout mice exhibit increased interscapular brown adipose tissue mass, the enhanced browning of white adipose tissue, and elevated thermogenesis [104], suggesting that this gene may play a role in cold adaptation by modulating energy expenditure and thermogenesis.

Additionally, Yudin et al. [105] employed hapFLK and DCMS methods to analyze candidate regions in the genomes of native Russian cattle and sheep breeds. Their analysis identified 31 genes crucial for the adaptation of these species, as well as other mammals, to the cold Arctic environment. Notably, the *NEB* gene was found in positively selected regions of the genomes of cattle, sheep, mammoths, polar bears, and whales. This gene is implicated in the regulation of heat production through shivering thermogenesis, a key process for survival and reproduction in extreme cold environments. A summary of potential candidate genes associated with cold adaptation is presented in Table 2.

### 3.3. Genes Linked to Adaptations to Heat Environments inHerbivorous Livestock

In the context of global warming, animal husbandry is confronting significant challenges, particularly the direct effects of elevated temperatures on animal health, survival, and productivity. As climate change intensifies, understanding the underlying molecular mechanisms that govern heat tolerance in livestock becomes crucial. Investigating the genetic, physiological, and biochemical pathways involved in heat adaptation will be essential to developing strategies for improving the resilience of animals in increasingly hot environments. Notably, Akinsola et al. [106] identified key regions of homozygosity (ROHs) associated with several genes, including *RIMS1*, *RSAD2*, *CMPK2*, *NOTCH1*, *OR1J1*, and *SLC25A17*. These genes are implicated in various biological processes, such as immune response, cellular stress mechanisms, olfactory function, and metabolism. Evidence suggests these genetic factors may confer inherent adaptability and disease resistance to extreme environmental stressors experienced by breeds in sub-Saharan Africa. Additionally, investigations revealed that the *HIPK1* gene, which regulates stress responses, potentially enhances heat stress resilience in Kuri cattle. Similarly, 753 differentially expressed genes (DEGs) were identified between heat-tolerant and non-heat-tolerant buffalo [107]. Weighted gene co-expression network analysis (WGCNA) identified modules associated with heat stress indices. The turquoise module demonstrated the most significant positive correlation with respiratory rate (RR) and rectal temperature (RT) and was notably enriched. Six genes involved in cytokine–cytokine receptor interactions (*IL18RAP*, *IL6R*, *CCR1*, *PPBP*, *IL1B*, and *IL1R1*) were identified as key hub genes, providing crucial insights into heat tolerance mechanisms in buffalo [107]. In complementary research, Luo et al. [108] employed weighted single-step genome-wide association study (WssGWAS), RNA sequencing, and prior findings to identify candidate genes associated with heat stress in heat-tolerant Chinese Holstein cattle. These genes—*PMAIP1*, *SBK1*, *TMEM33*, *GATB*, *CHORDC1*, *RTN4IP1*, and *BTBD7*—correlated with three physiological indicators of heat stress: rectal temperature, respiration rate, and drooling score. Under thermal challenge conditions, heat-tolerant Chinese Holstein cattle exhibited significantly (*p* < 0.05) attenuated elevations in rectal temperature and respiration rate, with reduced milk yield decline compared to non-heat-tolerant counterparts. Conversely, plasma concentrations of heat shock proteins (*HSP70* and *HSP90*) and cortisol were significantly (*p* < 0.05) elevated in heat-tolerant animals, underscoring enhanced stress response mechanisms. Furthermore, transcriptomic analysis revealed the significant enrichment of genes including *OAS2*, *MX2*, *IFIT5*, and *TGFB2*, which participate in immunomodulatory pathways contributing to thermal tolerance [109]. Additionally, Li et al. [110] conducted a study on the heat tolerance of Dehong humped cattle, a breed known for its exceptional heat resistance in Southwest China. They identified a series of candidate genes associated with heat shock response (*HSF1*), oxidative stress (*PLCB1*, *PLCB4*), coat color (*RAB31*), feed intake (*ATP8A1*, *SHC3*), and reproduction (*TP63*, *MAP3K13*, *PTPN4*, *PPP3CC*, *ADAMTSL1*, *SS18L1*, *OSBPL2*, *TOX*, *RREB1*, and *GRK2*). These genes positively influence the heat tolerance of Dehong humped cattle, enabling them to thrive in tropical climates. Consistently, Wang et al. [111] investigated the allele frequency distribution of missense mutations in the *EIF2AK4* gene across various Chinese native cattle breeds with differing geographic distributions. Through correlation analyses between genotypes and environmental factors, they identified significant geographic variation in the *EIF2AK4* gene variants. Specifically, the frequency of the mutant allele G showed a progressive increase from northern to southern cattle populations, with the highest frequency observed in herds from the southeastern regions, particularly those exposed to higher ambient temperatures [111]. Furthermore, a study included Angus and Simmental cattle, categorizing individuals within these breeds as either poorly adapted or well adapted to heat stress [112]. Their results indicated that respiratory rate and rectal temperature were effective indicators of heat tolerance. Additionally, the expression levels of the *HSF1* and *HSPA6* genes varied according to acclimatization status, with poorly acclimatized animals exhibiting elevated expression levels. These findings suggest that both genes may serve as biomarkers for heat tolerance in taurine cattle [112]. Subsequently, Pires et al. [113] assessed the expression levels of genes within the heat shock protein (HSP) family, including *HSPD1*, *HSPA1A*, and *HSP90AA1*, in two tropical cattle breeds, Nelore and Caracu. Their findings revealed a significantly higher expression of *HSPD1* in Caracu compared to Nelore. In contrast, *HSP90AA1* was more highly expressed in Nelore. No significant differences were observed in the expression of *HSPA1A* between the two breeds, suggesting breed-specific variations in the expression of heat shock proteins in response to thermal stress. Furthermore, a study investigated the heat stress response in a population of Gir × Holstein F2 crossbred cattle through a GWAS [114]. They employed gene-transcription factor (TF) network and Gene Ontology (GO) enrichment approaches to identify genes implicated in biological processes associated with heat stress. The candidate genes identified, including *LIF*, *OSM*, *TXNRD2*, and *DGCR8*, offer valuable insights into the molecular mechanisms underlying the response of dairy cattle to heat stress [114]. Building on prior GWAS findings, Jia et al. subsequently identified four novel SNPs in the *MYO1A* gene, with the frequency of these mutant alleles increasing from northern to southern Chinese cattle populations [115]. Additionally, significant correlations were observed between four annual climate indicators—temperature, relative humidity, temperature–humidity index, and average annual sunshine hours—suggesting their influence on the genetic response to heat stress [115].

In another experimental study focusing on potential heat resistance candidate genes in the skin of Santa Ines sheep, the results indicated upregulation in the expression of cell protection genes (*HSPA1A* and *HSPA6*) and immune response genes (*CXCL1*, *CAPN14*, *SAA4*, and *IGHG4*). These findings suggest that these genes may play a role in enhancing cellular protection and boosting immunity in response to heat stress [116]. Similarly, Saadatabadi et al. [117] performed a comparative analysis of whole-genome sequencing data from local sheep populations in both cold and hot regions of Iran, identifying key genes such as *SIK2*, *FER*, *TLR4*, *ATP1A1*, and *CDK5RAP3*, which are involved in heat stress response-related pathways. These genes are critical for immune system function and heat tolerance. Additionally, they reported genes such as *CD109*, *CR2*, *EOMES*, and *MARCHF1*, as well as *HTR4*, *ALDH1A3*, and *TRHDE*, which are implicated in mitigating the heat shock response through the regulation of digestion and energy metabolism. Consistently, Ibrahim et al. [118] conducted a study identifying multiple SNPs in heat resistance-related genes in Barki and Aboudeleik lambs, specifically in *HSP90AB1*, *HSPB6*, *HSF1*, *STIP1*, and *ATP1A1*. Furthermore, they found an association of polymorphisms in these genes with phenotypic traits such as sheep skin temperature, respiratory frequency, and red blood cell count. Similarly, a recent study by Aboul-Naga et al. [119] examined the correlation between heat stress phenotypes and genome-wide SNPs in a sample of 206 sheep from three Egyptian breeds across six distinct geographical locations. Through GWAS analysis, they identified 46 SNPs, particularly those linked to pigmentation. Notably, SNPs in four genes—*MYO5A*, which is associated with calmness; PRKG1, which regulates body temperature; *GSTCD*, related to the respiratory system; and *RTN1*, involved in endoplasmic reticulum stress—were significantly correlated with heat tolerance [119]. In a subsequent investigation, Afsal et al. [120] analyzed the expression patterns of *HSP70* genes in several key organs of Malabari goats residing in the hot and humid regions of Southern India. Their findings indicated that heat stress significantly increased *HSP70* expression in the adrenal gland, while a notable decrease was observed in the thyroid and mesenteric lymph nodes. The authors proposed that the *HSP70* gene is both tissue-specific and function-specific, making it an ideal biomarker for assessing goat heat stress responses. Furthermore, Wang et al. [80] conducted a comprehensive analysis of the genetic characteristics of five local sheep breeds in China, identifying the *DNAJB5* gene in Duolang sheep, which demonstrates substantial resistance to heat stress. Their discovery enhances the understanding of the genetic adaptations of sheep to hot environments and provides new molecular insights into their behavioral traits. Li et al. [121] further investigated the effects of acute heat stress on Hu Sheep, performing a transcriptome analysis of hypothalamic tissue to identify 1424 differentially expressed genes. These findings showed significant alterations in pathways related to calcium signaling, ribosome biogenesis, apoptosis, and oxidative stress responses, offering insights into potential protective measures against heat stress in livestock. A previous report conducted a comprehensive analysis of RNA sequencing data comparing a heat stress-treated sheep group with an untreated control group. This analysis revealed significant differences in key biological processes, including body temperature regulation (*5-HTR4* and *HTR1B*), stress response (the *Rap1*, *MAPK*, and PI3K-Akt pathways), energy metabolism (*NPR1*, *ANGPT2*, and *SLC13A5*), and immune response (*HSPA2*) [122]. Pantoja et al. [123] found that the expression levels of genes such as *GPX3*, *LOC101108817 (IGHG1)*, *VLDLR*, *EVC*, *GPAT3*, and *RGS6* in the livers of heat-tolerant sheep were significantly higher than those in non-heat-tolerant sheep when exposed to heat stress conditions (i.e., 36 °C). These genes were primarily enriched in the Hedgehog signaling pathway, glutathione metabolic process, glycerolipid metabolism, and thyroid hormone biosynthetic pathways. These biological pathways contribute significantly to ameliorating the deleterious effects of thermal stress, thereby enhancing heat tolerance capabilities in sheep [123]. A comparative analysis revealed that under heat stress conditions, Katjang goats maintained significantly lower rectal temperature (RT), hemoglobin (Hb), and packed cell volume (PCV) values relative to Boer goats, concomitant with elevated *HSP70* gene expression. This physiological profile strongly indicates superior thermotolerance in Katjang goats compared to their Boer counterparts [124].

The investigation of heat stress response in Brazilian horses identified significant genomic regions containing genes associated with redox processes (*ADO*, *GRHPR*, *GFOD1*, *KLF9*, *PIP5K1B*, *RANBP9*, and *JMJD1C*) and heat shock protein expression (*HSP40*, *HSP70*, *HSP27*, and *HSP90*) [125]. These findings elucidate the genetic mechanisms facilitating adaptation to tropical climatic conditions. Further genomic analysis utilizing whole-genome copy number variation (CNV) in Jinjiang horses, through *CNVR* gene annotation and quantitative PCR (q-PCR) methodology, identified and validated four pivotal genes—*NFKBIA*, *SOCS4*, *HSPA1A*, and *IL6—*that exhibit differential expression patterns under varying heat stress durations, suggesting their functional relevance in adaptation to high-temperature and high-humidity environments [126]. Table 3 presents a comprehensive overview of candidate genes implicated in heat tolerance across diverse livestock species.

### 3.4. Genes Linked to Adaptations to Drought Environments in Herbivorous Livestock

Arid and desert regions are characterized by extreme environmental conditions, including significant temperature fluctuations, severe water scarcity, intense ultraviolet radiation, and limited vegetation and water resources [127]. These harsh conditions pose significant challenges to the survival and productivity of organisms inhabiting such environments. To better understand how species adapt to these stresses, it is essential to investigate the molecular mechanisms underlying drought tolerance. A comprehensive understanding of these adaptive processes is critical for the conservation and sustainable management of genetic resources in endemic species, particularly those adapted to arid ecosystems. For instance, Lyu et al. [128] employed four selective scanning methods and identified several potential genes in Anxi cattle. These genes include *RBFOX2*, *CERS3*, *SLC16A7*, and *SPATA3R,* which are associated with heart development, cellular energy metabolism, enhancing the skin barrier, pancreatic endocrine function, and, reproductive capability, respectively. Collectively, these genes likely contribute to the remarkable ability of Anxi cattle to withstand extreme dry climatic conditions while maintaining normal production and reproduction. In parallel, Sukhija et al. [129] identified genes related to key physiological traits, including production performance (*CACNA1D* and *GHRHR*), reproduction (*ESR1* and *RBMS3*), immune response (*NOSTRIN* and *IL12B*), and environmental adaptation (*ADAM22* and *ASL*) using genome-wide selective marker analysis combined with CLR and Fst methods. These findings may contribute to better understanding the genetic characteristics of cattle breeds endemic to the arid regions of India. Consistently, Liu et al. [130] identified specific gene variants in drought-adapted Anxi cattle that are associated with several key physiological pathways, including arachidonic acid metabolism, the renin–angiotensin system, the oxytocin signaling pathway, and the pancreatic secretory pathway. These genetic adaptations may enhance the ability of cattle to survive and reproduce in conditions characterized by extreme desiccation, forage scarcity, and high soil salinity. Furthermore, Ben-Jemaa et al. [131] used SNP microarray data alongside four distinct genome selection scanning strategies to pinpoint *GH1*, a gene undergoing strong positive selection in North African cattle populations. This gene is strongly linked to several critical biological processes, such as nutrient-level response, the positive regulation of lactation activity, and triglyceride biosynthesis. These functions are thought to contribute to the adaptation of North African cattle to seasonal fluctuations in food resources typical of arid regions, thus enhancing their survival and reproductive success.

In the extremely arid environment of the Taklamakan Desert, native sheep exhibit body size regulation through genes such as *RAPSN* and *CNBD2*. These genes facilitate skin vasodilation for heat dissipation via *KCNJ16*, *KCNMB2*, and *PLCG1*, optimize renal function through *BMP7*, facilitate lens development for ultraviolet filtration via *CELF1*, and contribute to pigmentation through *TECRL*. Collectively, these genes may contribute to an efficient set of adaptive mechanisms, allowing these sheep to endure extreme arid conditions [31].

Similarly, Asadollahpour et al. [32] employed gene-selective scanning to show that *KITLG*, a gene involved in skin pigmentation, exhibits strong positive selection in Southwest Asian goats. This gene is thought to enhance adaptation to UV radiation and temperature fluctuations, thus improving the adaptive flexibility of these goats [132]. Consequently, Zhang et al. [133] identified candidate genes associated with immune response (*DNTT*, *FEN1*, *POLL* and *PRKDC*, etc.), visual correlation (*MAFB*, *PTEN*, *MITF* and *EDN3*, etc.), heat stress tolerance (*F13A1*), and high reproductive rates (*FGF3*, *ARNTL*, *CHKA* and *NRG4*, etc.) at a 1% threshold during a genome scan of native sheep in the Taklamakan Desert. These findings provide comprehensive information of the genetic mechanisms underlying the adaptation of sheep to desert environments. Similarly, a study investigating the genetic adaptations of three native Iranian sheep breeds to hot and arid conditions identified two key genes (*CORIN* and *CPQ*) that play significant roles in coping with extreme environmental stress [134]. The *CORIN* gene, which is closely involved in blood volume regulation, helps ensure adequate blood flow, thus supporting essential thermoregulatory processes critical for survival under heat stress [135]. Additionally, the *CPQ* gene, which contributes to protein hydrolysis, was found to be stable in these breeds, suggesting that drought conditions may not adversely affect the activity of protein-hydrolyzing enzymes. This stability could potentially enhance drought resistance in sheep by maintaining efficient protein turnover in the face of water scarcity [136]. Moreover, Chebii et al. [137] detected genomic selection signals in the Nubian ibex through a comparative analysis of protein-coding genes. The *ABCA12* and *ASCL4* genes, which are involved in skin barrier development, along with the DNA repair gene *UVSSA*, exhibited positive selection signals. This indicates that the Nubian ibex inhabiting hot desert environments have evolved skin protection strategies to minimize water loss and damage from solar radiation by enhancing the epidermal barrier system.

Furthermore, a genome-wide selection scan investigating the genetic basis of drought adaptation in Liangzhou donkeys revealed a significant selection signal for the *CYP4A11* gene, located on chromosome 5. This finding suggests that *CYP4A11* plays a crucial role in the adaptation of Liangzhou donkeys to arid conditions [138]. The *CYP4A11* gene encodes an enzyme involved in the conversion of arachidonic acid to 19(S)-HETE, a metabolite that is essential for promoting water reabsorption in the renal tubules [139]. By enhancing renal water retention, this mechanism helps Liangzhou donkeys minimize water loss during periods of drought, thus contributing to their physiological adaptation and survival in harsh, water-scarce environments. Table 4 provides a detailed summary of potential candidate genes associated with drought adaptation in livestock.

## 4. Suggestion and Recommendations

Based on the presented contents, we would like to offer some recommendations and suggestions. The practical application of these genetic markers offers significant benefits for farmers and government agencies. Farmers could implement marker-assisted selection programs to develop more resilient herds while maintaining productivity, potentially through the strategic crossbreeding of adapted indigenous breeds with high-producing commercial varieties. Governments should establish conservation programs for valuable indigenous genetic resources, subsidize genetic testing for small-scale farmers, and develop regional breeding centers focused on location-specific adaptations. These approaches would yield economic benefits through reduced mortality, enhanced productivity under stress conditions, lower management costs, and potential market premiums for climate-resilient livestock. These strategies collectively support sustainable livestock production amid increasing environmental challenges.

## 5. Conclusions

Climate change poses significant threats to livestock productivity and reproduction, driven by resource scarcity, disease prevalence, heat stress, and biodiversity loss, which collectively result in substantial economic losses [140,141,142,143]. While traditional breeding has prioritized production traits, the integration of genetic markers related to environmental resilience—such as those identified in our study—can accelerate the development of robust breeds through advanced techniques like whole-genome selection. Our findings highlight key genetic markers associated with environmental adaptation in herbivorous livestock, particularly in understudied species like horses and donkeys. These markers, linked to traits such as cold and heat tolerance, drought resistance, and high-altitude adaptation, provide a foundation for future breeding programs. However, the functional validation of these candidate genes remains a critical gap that must be addressed to fully harness their potential. In conclusion, our study underscores the importance of genetic research in enhancing livestock resilience to climate change. By leveraging genome-wide selection and focusing on candidate genes, we can develop sustainable breeding strategies that support both animal welfare and agricultural productivity. Future research should expand the scope of genetic studies across diverse species and environments while also emphasizing the functional validation of adaptive traits. Such efforts will be essential for ensuring the sustainability of global livestock production in the face of escalating environmental challenges.

## Figures and Tables

**Table 1:** Summary of potential genes associated with high-altitude adaptation in herbivorous livestock.

Species	Genes	Variations	Methods	Function	References
Yak	*CAMK2B*, *GLUL*	-	dN/dS, ω	Encode for key components in calcium signaling and nitrogen metabolism	[54]
Tibetan cattle	*EGLN1*	-	Fst, θπ, XP-EHH and Tajimas’ D	Hypoxia tolerance	[55]
Ethiopian indigenous cattle	*CLCA2* *SLC26A2 CBFA2T3*	-	iHs, XP-CLR and PBS	Renin secretion KEGG pathway Ion channel activity Response to hypoxia	[56]
Ladakhi cattle	*HIF-1A* *VPS13C*, *CPT1A* *DNAJA3*, *HSPA2*, *HSP90AB1* *PER1*, *CRY1*, *ARNTL*, *CLOCK*, *FBXL3*	-	Fst and π	Hypoxia adaptation Energy metabolism Cold adaptation Circadian rhythm	[57]
Gayal, Yak	*UQCRC1*, *COX5A* *CAPS* *EDN3* *CHRM2* *EGLN1*	-	edgeR	Related to the energy supply of myocardial contraction Related to pulmonary artery smooth muscle contraction Related to the tracheal epithelium and pulmonary vasoconstriction The autonomous regulation of the heart The target gene of the hypoxia-sensing pathway	[58]
Tibetan cattle	*ACSS2*	rs43717468 A > G, rs439295601 G > T in the 5′-flanking region	FLK, hapFLK, XP-EHH, and composite Fst	Promotes metabolic adaptation to hypoxia via the hypoxia-inducible factor (HIF) pathway	[59]
Yak	*HSPB7*, *HSPB2*, *HSPD1*, *HSPA1L*, *HSP90AA1* *VEGFA*	-	DESeq2, WGCNA	Encode the heat shock protein (HSP) protein family Maintain the balance of blood vessel density	[60]
Yak	*UVSSA*	-	clusterProfiler package in R	Resistance to UV radiation	[61]
Yak	*GRIK4*, *IFNLR1*, *LOC102275985*, *GRHL3*, *LOC102275713*	*GRIK4* (a dup CNV_202) *IFNLR1* (a dup CNV_265) *LOC102275985* (del CNV_199 and del CNV_200) *GRHL3* (a dup CNV_265) *LOC102275713* (a del CNV_201)	Fst, V_ST_	Physiological regulation under a hypoxic environment	[62]
Yak	*RPS6KA6*, *ITPR1*, *GNAO1*, *PDE4D*	-	Fst, ZHp	Associated with environmental adaptability	[63]
Yak	*EPAS1*	*-*	voom embedded in the R package limma	Encodes hypoxia-induced-factor 2α	[64]
Yak	*DCC*, *GSTCD*, *MRPS28*, *MOGAT2*	-	CNVnator	Adaptation to hypoxia	[65]
Pali yak	*MMP3*	rs2381 A > G and rs4331 C > G in intron V and intron VII	Haplotype analysis	Regulates the cellular response to hypoxia	[67]
Qaidam cashmere goat	*TH*, *ACER1*, *GNB1*, and *HIF1A*	-	Fst, θπ	Hypoxic adaptation	[68]
Tibetan goat	*LEPR*, *LDB1*, *EGFR* and *FGF2*	-	Fst	Associated with high-altitude adaptation	[69]
Ethiopian indigenous goat	*PTPMT1*	-	Hp, Fst, XP-EHH	Associated with critical hypoxic survival gene	[70]
Tibetan goat	*ADIRF*	-	Fst, XP-EHH	Relates to high-altitude adaptation	[72]
Tibetan goat	*PAPSS2*	chr26: 42012872 *G* > T in intron region	Fst, XP-EHH	Adaptation to hypoxia	[73]
Nepalese goat	*FGF5*, *EPAS1*	*FGF5* (c.-253G>A within 5’UTR) *EPAS1* (Q579L in exon 5)	Pearson correlation coefficients	Regulated hair growth; encodes hypoxia-induced-factor 2α	[36]
Tibetan sheep	*EPAS1* *PAPSS2* *PTPRD*	*EPAS1* (1 deletion SV) *PAPSS2* (1 insertion SV) *PTPRD* (1 deletion SV)	Delly, Manta, Amoove	High-altitude adaptation Blood circulation Pulmonary hypertension	[75]
Tibetan sheep	*HBBC*, *HBB*, *EGLN1*	*EGLN1* (Chr25:3503284 *G* > A in 3′UTR*)*	XP-EHH, XP-CLR	Relates to high-altitude adaptation	[76]
Tibetan sheep	*HIF-1α*	g.76805181 *G* > A in exon 9, g.76806025 *G* > A in exon 10, and g.76808146 T > A in exon 12	Haplotype analysis	Key regulator of adaptation to high-altitude hypoxia	[77]
White Tibetan sheep	*HIF1A*, *ATR*, *SLC24A*, *PPA2*, *ROCK2*	-	Fst, XP-EHH	Linked to high-altitude adaptation	[78]
Tibetan sheep	*HAG1*	C-INDEL in 5′UTR	Fst, ZHp	Relates to high-altitude adaptation	[79]
Tibetan sheep	*CYP17*	-	Fst, Hp	Associated with hemoglobin levels	[80]
Ganzi horse	*EPAS1*, *ABTB2*, *RHOQ*, *TMEM247*	*EPAS1 (*Chr15:53538574 A *>* T)	CLR, iHS, Fst, XP-EHH	Related to high-altitude adaptation	[82]
Tibetan Horses	*EPAS1*	*SNP1 (R144C)*, *SNP2 (E263D)*	Fst, ZHP, θπ	Encodes hypoxia-induced-factor 2α	[53]
Equus kiang	*GH43*, *GH3*, *GH31*, *GH5*, *GH10*	-	metastat analysis	Associated with carbohydrate metabolism genes	[83]
Kiang	*EPAS1*	*-*	Fst, θπ	Encodes hypoxia-induced-factor 2α	[84]
Tibetan donkeys	*EGLN1*	*-*	Fst, θπ	Associated with high-altitude adaptation	[84]
Qinghai donkey	*HBB*, *GLDC*	*-*	ZH_P_	Relates to high-altitude adaptation	[87]

**Table 2:** Potential genes associated with cold adaptation in herbivorous livestock.

Species	Genes	Variations	Methods	Function	References
Chinese native cattle	*ZC3H10*	-	FLK and hapFLK	Participates in thermogenesis and immune responses	[88]
Cross-bred cattle	*TRPM8*, *NMUR1*, *OXR1*, *PRKAA2*, *SMTNL2* *PCLB4*, *SIN3A*	*-*	*θ*π, XP-CLR, Fst	Metabolic homeostasis Immune responses	[89]
Chinese indigenous cattle	*UQCR11*, *DNAJC18*, *EGR1*, *STING1*	*UQCR11* (rs43485110 C > T and rs110122520 C > G in the 5’-flanking region) *DNAJC18* (rs207746463 C > T in the 5′-flanking region) *EGR1* (c.190 G > A SNP) *STING1* (c.601 C > A SNP)	hapFLK, FLK	Hermogenesis and energy metabolism	[90]
Northern cattle	*PRDM16*	c.2336 T > C, p.L779P SNV	Fst, Pi, Tajima’s D	Maintains brown adipocyte formation	[91]
Yakut cattle	*NRAP*	*Chr26:34131393* G > T SNP	hapFLK	Associated with myofibrillar assembly and force transmission in the heart	[92]
Yanbian Cattle	*CORT* *FGF5* *CD36*	*CORT* (c.269C > T, p.Lys90Ile; c.251A > G, p.Glu84Gly; c.112C > T, p.Pro38Ser; c.86G > A, p.Pro29His) *FGF5* (c.191C > T, p.Ser64Phe) *CD36* (c.638A > G, p.Lys 213Arg)	*θ*π, XP-CLR, Fst	Regulate primary hormone in the hypothalamic–pituitary–adrenal (HPA) axis Linked with hair follicle and length development Affect intramuscular fat deposition	[93]
Swedish cattle	*AQP3*, *AQP7*, *HSPB8*	-	Tajima’s D, Pi, Fst	Associated with cold adaptation	[94]
Inner Mongolia Sanhe cattle	*Hsp70*	SNP-42^−^ C > T, SNP-205 ^+^ G > T	General linear model procedure and Bonferroni *t* test	Cytoprotective effects by regulating pathways related to cell stress response	[95]
Eastern Finncattle	*DNAJC28*, *HSP90B1*, *AGTRAP*, *TAF7*, *TRIP13*, *NPPA*, *NPPB*	-	SweeD, CLR	Associated with cold adaptation	[96]
Western Finncattle	*CD14*, *COBL*, *JMJD1C*, *KCNMA1*, *PLA2G4*, *SERPINF2*, *SRA1*, *TAF7*	-	SweeD, CLR	Boost up adaptability to cold environment	[96]
Yakutian cattle	*DNAJC9*, *SOCS3*, *TRPC7*, *SLC8A1 GLP1R*, *PKLR*, *TCF7L2*	-	SweeD, CLR	Enhance resistance to cold weather	[96]
Siberian cattle	*MSANTD4*, *GRIA4*	-	H1, H12, Pi, DCMS, Tajima’s D	Indirect involvement in the cold shock response and body thermoregulation	[97]
Altay sheep, Hu lambs	*APOC3*, *FABP4*, *LPL*, *PCK1*, *ADCY10*, *ADORA2A*, *MYL2*	-	Pairwise comparisons	Thermoregulation and muscle contraction	[98]
Altay lambs	*ACTA1*, *MYH1*, *MYH2*, *MYL1*, *MYL2*, *TNNC1*, *TNNC2*, *TNNT3* *ATP2A1*, *SLN*, *CKM*	-	DESeq2 software (https://bioconductor.org/packages/release/bioc/html/DESeq2.html (accessed on 17 January 2025))	Muscle shivering thermogenesis Muscle non-shivering thermogenesis	[99]
Altay lambs, Hu lambs	*UCP1*, *RYR1*, *ADIPOQ* and *LPL*	-	DESeq2 software	Thermogenesis	[100]
Changthangi sheep	*TRPM8*	-	ROH, iHS	Enhances ability of animals to tolerate cold weather	[101]
Kulundin, Altai Mountain, and Baikal sheep	*ADAMTS5*	-	hapFLK, *p*-value, DCMS, Tajima’s D, F_ST_, Pi	Promotes adipogenesis and white adipose tissue expansion	[103]
Russia sheep and cattle	*NEB*	-	hapFLK, DCMS	Thermoregulation	[105]

**Table 3:** Summary of potential genes linked with heat adaptation herbivorous livestock.

Species	Genes	Variations	Methods	Function	References
SSA cattle	*RIMS1*, *RSAD2*, *CMPK2*, *NOTCH1* *OR1J1* *SLC25A17*	-	iHS, EHH	Immune response, cellular stress mechanisms Olfactory function Metabolic efficiency	[106]
Water buffalo	*IL18RAP*, *IL6R*, *CCR1*, *PPBP*, *IL1B*, and *IL1R1*	-	WGCNA	Cytokine–cytokine receptor interaction	[107]
Chinese Holstein cattle	*PMAIP1* *SBK1* *TMEM33*, *GATB*, *BTBD7*, *CHORDC1*, *RTN4IP1*,	-	WssGWAS	Regulate diverse cellular functions in autophagic cell death and metabolism Involved in the protection of cells Heat stress/heat shock and cellular adaptive function	[108]
Chinese Holstein dairy cows	*OAS2, MX2, IFIT5* and *TGFB2*	-	DESeq2 R package	Activate immune effector process	[109]
Dehong humped cattle	*HSF1* *PLCB1*, *PLCB4* *RAB31* *ATP8A1*, *SHC3* *TP63*, *MAP3K13*, *PTPN4*, *PPP3CC*, *ADAMTSL1*, *SS18L1*, *TOX*, *RREB1*, *GRK2*, *OSBPL2*	-	Fst, XP-EHH, XP-CLR	Heat tolerance Oxidative stress response Coat color Feed intake Reproduction	[110]
Southern Chinese cattle	*EIF2AK4*	g.35615224 T > G in exon 6	POPGENE software (version 7.32)	Thermal stress	[111]
Angus and Simmental cattle	*HSF1*, *HSPA6*	-	RT-qPCR	Thermotolerance indicators	[112]
Nelore and Caracu beef cattle	*HSPD1*, *HSP90AA1*	-	qPCR	Enhance immune response, stress adaptation and heat tolerance	[113]
Gir × Holstein F2 cattle	*LIF*, *OSM*, TXNRD2, *DGCR8*	-	GWAS	Heat stress effects	[114]
Chinese indigenous cattle	*MYO1A*	g.56383560G > A, g.56383565T > C, g.56383578T > C, g.56383635A > G	PCR	Heat tolerance	[115]
Santa Ines sheep	*HSPA1A*, *HSPA6* *CXCL1*, *CAPN14*, *SAA4*, *IGHG4*	-	dgeR package	Cellular protection Promote immune response	[116]
Iranian sheep	*SIK2*, *FER*, *TLR4*, *ATP1A1*, *CDK5RAP3* *CD109*, *CR2*, *EOMES*, *MARCHF1* *HTR4*, *ALDH1A3*, *TRHDE*	*-*	Fst, θπ	Heat stress Immune response Control digestive metabolism	[117]
Barki and Aboudeleik lambs	*HSP90AB1*, *HSPB6*, *HSF1*, *ST1P1* and *ATP1A1*	*HSP90AB1* (3 SNPs:A118*G*, A478G, C232T) *HSPB6* (1 SNP:C155T) *HSF1* (3 SNPs:G283A, G170A, C410T) *ST1P1* (4 SNPs:A177*G*, C336T, C491T, A457) *ATP1A1* (2 SNPs:A47*G*, C143T)	PCR	Heat tolerance	[118]
Egyptian sheep	*MYO5A* *PRKG1* *GSTCD* *RTN1*	*MYO5A* (Chr7:54927841 G > A, Chr7:54966248 A > C) *PRKG1* (Chr22:8045910 G > A) *GSTCD* (Chr6:19441931 G > A) *RTN1*(Chr7:68761822 G > A)	statgenGWAS	Pigmentation Body thermoregulation Respiratory system Alleviate endoplasmic reticulum stress	[119]
Malabari goats	*HSP70*	-	RT-PCR	Heat stress acclimation	[120]
Duolang sheep	*DNAJB5*	*-*	Fst, Hp	Heat tolerance	[80]
Hu sheep	*5-HTR4*, *HTR1B* *NPR1*, *ANGPT2,* *SLC13A5* *HSPA2*	-	DESeq2 software	Body thermoregulation Energy metabolism Immune response	[122]
Thermotolerant sheep	*GPX3* *IGHG1* *VLDLR* *EVC* *GPAT3* *RGS6*	-	EdgeR	Protect cells from heat damage Immunoglobulin synthesis Body temperature regulation Intercellular communication Metabolic homeostasis Cellular signaling and responses to heat stress	[123]
Brazilian horse	*ADO*, *GRHPR*, *GFOD1*, *KLF9*, *PIP5K1B*, *RANBP9*, *JMJD1C HSP40*, *HSP70*, *HSP27*, *HSP90*	-	hapFLK, PCAdapt	Oxidative reduction Encode heat shock protein	[125]
Jinjiang horse	*NFKBIA* *SOCS4* *HSPA1A* *IL6*	-	qPCR	Inhibit heat shock Negatively regulate the inflammatory response Protect cell homeostasis Regulate the immune system	[126]

**Table 4:** Summary of potential genes associated with drought adaptation in livestock.

Species	Genes	Variations	Methods	Function	References
Anxi cattle	*RBFOX2* *CERS3* *SLC16A7* *SPATA3*	*SPATA3* (c. 184G>A, rs43347904)	CLR, F_ST_, θπ, XP-CLR	Cardiac development Involved in regulating skin permeability and antimicrobial functions Regulation of pancreatic endocrine function Reproduction	[128]
Indian cattle	*CACNA1D*, *GHRHR ESR1*, *RBMS3* *NOSTRIN*, *IL12B* *ADAM22*, *ASL*	-	CLR, F_ST_	Production traits Reproduction Immunity Environmental adaptation	[129]
Zhangmu cattle, Qaidam cattle, Anxi cattle, Kazakh cattle, Mongolian cattle	*PLA2G4B*	-	θπ, F_ST_, Tajima’s D	Regulates water retention and reabsorption	[130]
North African cattle	*GH1*	-	iHS, Rsb, XP-EHH, F_ST_	Responds to nutrient levels, positive regulation of lactation and triglyceride biosynthetic process	[131]
Taklamakan Desert sheep	*RAPSN*, *CNBD2* *KCNJ16*, *KCNMB2* *PLCG1*, *BMP7* *CELF1* *TECRL*	-	XP-EHH, iHS, F_ST_	Body sizes Facilitate heat dissipation Kidney function and development Lens development Pigment deposition	[31]
Southwest Asia goats	*KITLG*	-	θπ, F_ST_, Tajima’s D	Pigmentation	[32]
Taklamakan Desert sheep	*DNTT*, *FEN1*, *POLL PRKDC* *MAFB*, *PTEN*, *MITF EDN3 F13A1* *FGF3*, *ARNTL*, *CHKA* *NRG4*	-	F_ST_, XP-EHH, Rsb, iHS	Immunity Vision Heat stress tolerance High reproduction rate	[133]
Iranian sheep	*CORIN*, *CPQ*	-	F_ST_, Pi	Maintain the proper volume of blood and proteolytic functions	[134]
Nubian ibex	*ABCA12*, *ASCL4*, *UVSSA*	-	comparative analysis of protein-coding genes	Skin barrier development and DNA repairing	[13]
Liangzhou donkey	*CYP4A11*	*-*	F*_ST_,* θπ, CLR, XP-EHH	Promotes water reabsorption	[138]

## Abbreviation

The following abbreviations are used in this manuscript:

F_ST_	Fixation index
π	Nucleotide diversity
XP-EHH	Cross-population extended haplotype homozygosity
Tajimas’ D	Tajima’s D statistic
iHs	Integrated haplotype score
XP-CLR	Cross-Population Composite Likelihood Ratio
PBS	Population Branch Statistic
edgeR	Empirical Analysis of Digital Gene Expression Data in R
FLK	Fixation index of Lewontin and Krakauer
hapFLK	Haplotype Fixation index of Lewontin and Krakauer
DESeq2	Differential gene expression analysis based on the negative binomial distribution
WGCNA	Weighted gene co-expression network analysis
V_ST_	Variance stabilizing transformation
ZHp	Z-transformed haplotype homozygosity
Hp	Haplotype homozygosity
CLR	Composite likelihood ratio
iHS	Integrated haplotype score
Pi	Nucleotide diversity
SweeD	Sweep detector
DCMS	De-correlated composite of multiple signals
ROH	Run of homozygosity
EHH	Extended haplotype homozygosity
WssGWAS	Weighted single-step genome-wide association study
RT-qPCR	Reverse transcription quantitative polymerase chain reaction
qPCR	Quantitative polymerase chain reaction
PCR	Polymerase chain reaction
GWAS	Genome-wide association study
Rsb	Ratio of shared branch lengths
